# Increased CXCR3 Expression of Infiltrating Plasma Cells in Hunner Type Interstitial Cystitis

**DOI:** 10.1038/srep28652

**Published:** 2016-06-24

**Authors:** Yoshiyuki Akiyama, Teppei Morikawa, Daichi Maeda, Yukako Shintani, Aya Niimi, Akira Nomiya, Atsuhito Nakayama, Yasuhiko Igawa, Masashi Fukayama, Yukio Homma

**Affiliations:** 1 Department of Urology, Graduate School of Medicine, The University of Tokyo, Tokyo, Japan; 2 Department of Continence Medicine, Graduate School of Medicine, The University of Tokyo, Tokyo, Japan; 3 Department of Pathology, Graduate School of Medicine, The University of Tokyo, Tokyo, Japan; 4 Department of Cellular and Organ Pathology, Graduate School of Medicine, Akita University, Akita, Japan; 5 Department of Urology, Mitsui Memorial Hospital, Tokyo, Japan

## Abstract

An up-regulated CXCR3 pathway and affluent plasma cell infiltration are characteristic features of Hunner type interstitial cystitis (HIC). We further examined these two features using bladder biopsy samples taken from 27 patients with HIC and 15 patients with non-IC cystitis as a control. The number of CD3-positive T lymphocytes, CD20-positive B lymphocytes, CD138-positive plasma cells, and CXCR3-positive cells was quantified by digital image analysis. Double-immunofluorescence for CXCR3 and CD138 was used to detect CXCR3 expression in plasma cells. Correlations between CXCR3 positivity and lymphocytic and plasma cell numbers and clinical parameters were explored. The density of CXCR3-positive cells showed no significant differences between HIC and non-IC cystitis specimens. However, distribution of CXCR3-positivity in plasma cells indicated co-localization of CXCR3 with CD138 in HIC specimens, but not in non-IC cystitis specimens. The number of CXCR3-positive cells correlated with plasma cells in HIC specimens alone. Infiltration of CXCR3-positive cells was unrelated to clinical parameters of patients with HIC. These results suggest that infiltration of CXCR3-positive plasma cells is a characteristic feature of HIC. The CXCR3 pathway and specific immune responses may be involved in accumulation/retention of plasma cells and pathophysiology of the HIC bladder.

Interstitial cystitis (IC) is a chronic bladder disease characterized by lower urinary tract symptoms such as urinary frequency, nocturia, urgency, and/or bladder pain, causing a deterioration in the sufferer’s quality of life^1^. To date, the pathophysiology of IC is largely unknown, although deficient barrier function of the urothelium, aberrant microvasculature, and neurogenic inflammation in the bladder have been suggested^2^,^3^,^4^,^5^.

IC can be classified into multiple distinguishable phenotypes, with Hunner type IC (HIC), by the presence of the Hunner lesions on cystoscopy^1^,^6^. Histologically, HIC is a distinct inflammatory disease characterized by predominant infiltration of lymphoplasmacytic cells and denudation of the urothelium among IC^7^,^8^,^9^. We have examined these features by quantitative evaluation of cell numbers using novel image analysis software, confirming the accumulation of plasma cells in the lamina propria of the HIC bladder^9^. Furthermore, we have found a light-chain restriction of plasma cells in HIC cases, which suggests clonal expansion of B cells and possible involvement of immune responses in the persistent inflammation of HIC^9^. On the other hand, HIC is associated with up-regulated gene expression of CXCR3, a receptor for proinflammatory chemokines such as CXCL9, CXCL10, and CXCL11^10^,^11^. The CXCR3 pathway plays a crucial role in the persistent chronic inflammation seen, for example, in allergic and autoimmune diseases, because of its major chemoattractant properties in recruitment of inflammatory cells^12^,^13^,^14^,^15^.

Here, to further characterize the inflammatory reaction in HIC, we examined CXCR3 expression of infiltrating immune cells in HIC specimens by immunohistochemistry using non-IC cystitis specimens as a control.

## Results

### Study population

Demographics and characteristics in patients with HIC are shown in Table 1. Gender distribution showed significant female predominance in HIC group (24 versus 3) compared with non-IC cystitis group (7 versus 8) (*p* < 0.01). The mean age was 68.4 (range 38–88) and 72.5 (range 54–85) years in HIC and non-IC cystitis groups, respectively (*p* = 0.26). The control non-IC cystitis patients underwent bladder biopsy under suspicion of bladder cancer, and were all free from bladder pain, bladder discomfort or urinary frequency. Of 23 control specimens, 12 were taken from non-cancerous areas of 8 patients with non-muscle invasive bladder cancer and 11 were from 7 patients without evidence of malignancy.

Conventional histological assessment of hematoxylin and eosin (H&E)-stained slides confirmed the presence of infiltrating inflammatory cells in all the specimens, with lymphoplasmacytic cells outnumbering granulocytes (eosinophils and neutrophils).

### Quantification of the cell number by image analysis software

**Figure 1:**
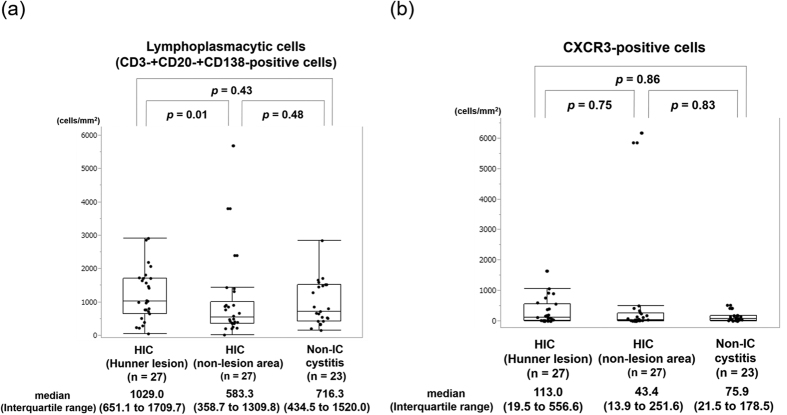
Quantification of lymphoplasmacytic cells and CXCR3-positive cells by image analysis software. (**a**) The number of lymphocytes and plasma cells in HIC and non-IC cystitis specimens. (**b**) The number of CXCR3-positive cells in HIC and non-IC cystitis specimens. Values are expressed as median (Interquartile range).

The number of lymphoplasmacytic cells or CXCR3-positive cells showed no significant differences between HIC and non-IC cystitis (Fig. 1). The number of CXCR3-positive cells significantly correlated with that of CD3-positive T cells or lymphoplasmacytic cells in all of the groups. Demographics and the number of CXCR3-positive, CD3-positive, CD20-positive and CD138-positive cells were not different between controls associated with bladder cancer and those without (Supplementary Table S1). Meanwhile, the number of CXCR3-positive cells significantly correlated with that of CD20-positive B cells or CD138-positive plasma cells in HIC specimens but not in non-IC cystitis control specimens (Table 2). The lack of correlation was observed regardless of the presence or absence of bladder cancer (Supplementary Table S2).

### Distribution patterns of CD138-and CXCR3-positivity

**Figure 2:**
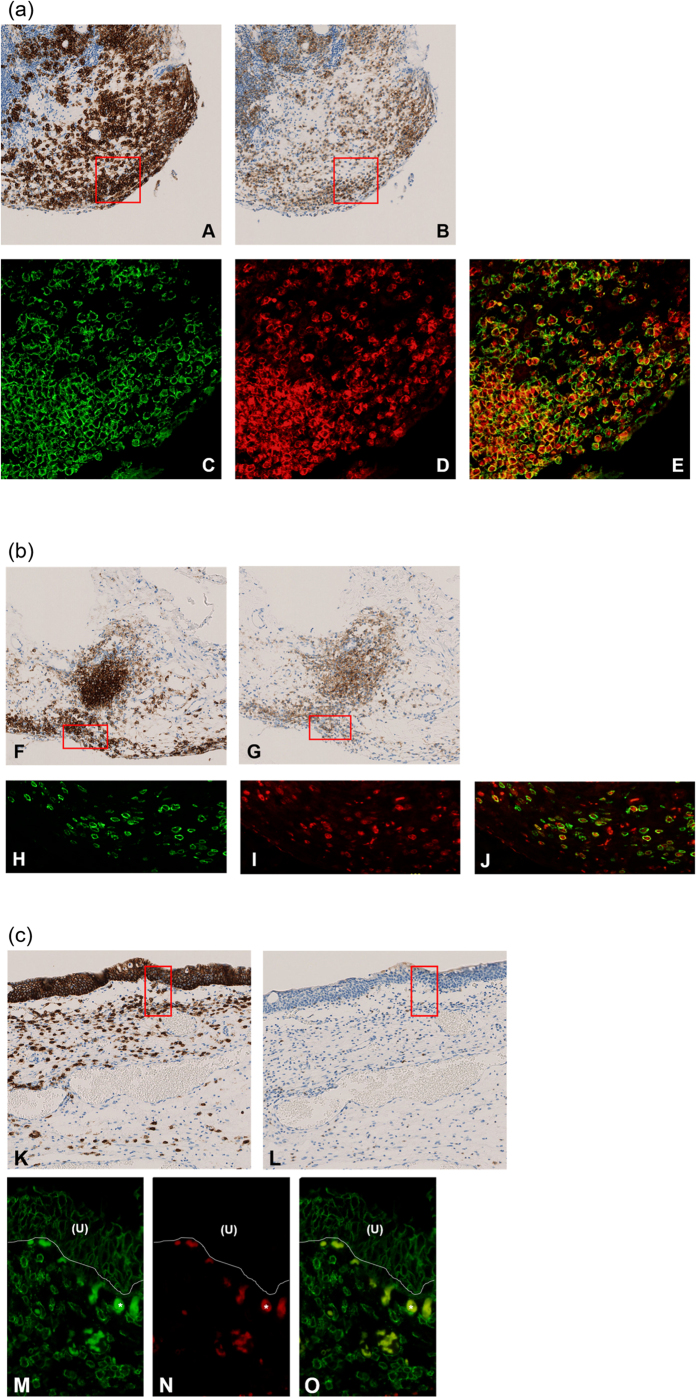
Representative images of localization of CD138-positive cells and CXCR3-positive cells. (**a**) HIC (Hunner lesion). (A and B) Consecutive sections from biopsies of the Hunner lesion in HIC stained with the antibodies for CD138 (A) and CXCR3 (B), respectively. (All original magnification, ×100). Please note the similar distribution of plasma cells (CD138-positive cells) and CXCR3-positive cells. (C, D and E) Double-immunofluorescence for CD138 (C, green) and CXCR3 (D, red) in the area outlined in the rectangular box in Fig. 2a. Merge of green and red is shown in panel E. (All original magnification, ×400). (C) CD138-positive cells (D) CXCR3-positive cells in corresponding images to C. Please note that most plasma cells (CD138-positive cells) co-express CXCR3. (**b**) HIC (non-lesion area). (F and G) Consecutive sections from biopsies of non-lesion areas in HIC stained with the antibodies for CD138 (F) and CXCR3 (G), respectively. (All original magnification, ×100). Please note the similar distribution of plasma cells (CD138-positive cells) and CXCR3-positive cells. (H, I and J) Double-immunofluorescence for CD138 (H, green) and CXCR3 (I, red) in the area outlined in the rectangular box in Fig. 2b. Merge of green and red is shown in panel J. (All original magnification, ×400). (H) CD138-positive cells (I) CXCR3-positive cells in corresponding images to H. Please note that most plasma cells (CD138-positive cells) co-express CXCR3. (**c**) Non-IC cystitis. (K and L) Consecutive sections from a biopsy in a non-IC cystitis case stained with the antibodies for CD138 (K) and CXCR3 (L). (All original magnification, ×100). Please note the different distribution of plasma cells (CD138-positive cells) and CXCR3-positive cells. (M, N and O) Double-immunofluorescence for CD138 (M, green) and CXCR3 (N, red) in the area outlined in the rectangular box in Fig. 2c. Merge of green and red is shown in panel O. (All original magnification, ×400). (M) CD138-positive cells (N) CXCR3-positive cells in a corresponding image to M. Please note few plasma cells (CD138-positive cells) co-express CXCR3. (U): Urothelium; *Non-specific staining of red blood cells for CD138 and CXCR3.

Relationship of CD138-positive cells and CXCR3-positive plasma cells in HIC was examined in plasma cell-rich areas, which were identified in 22 of 27 HIC specimens and 8 of 15 non-IC cystitis specimens. Distribution of CD138-positive cells corresponded largely to that of CXCR3-positive plasma cells in HIC (Fig. 2a-A,B,b-F,G). The mean sum of proportion of the number of CXCR3-and CD138-positive cells to the number of all mononuclear cells was significantly higher in HIC cases than non-IC cystitis cases, and exceeded 1.0, suggesting the presence of CXCR3-and CD138-double positive cells (Table 3). Co-localization of CXCR3 positivity in CD138-positive plasma cells was confirmed by double-immunofluorescence staining (Fig. 2a-C,D,E,b-H,I,J). On the other hand, the distribution of CXCR3-positive cells and CD138-positive plasma cells was poorly matched in non-IC cystitis (Fig. 2c).

### Correlation between cell numbers and clinical parameters in HIC

No significant correlations between CXCR3-postive cells and any of the clinical parameters examined were observed (Table 4).

## Discussion

In the present study, we demonstrated that (1) the number or density of CXCR3-positive cells showed no significant differences between HIC and non-IC cystitis specimens; and (2) the majority of accumulating plasma cells expressed CXCR3 in HIC specimens, but not in non-IC cystitis specimens.

Lack of difference in the density of CXCR3-positive cells between IC and non-IC specimens may be contradictory to a previous report^11^, which indicated increased mRNA expression of genes related to the CXCR3 pathway in the IC bladder. This discrepancy could be explained by the difference in the comparative control; the control used in the previous study was ‘normal’ samples with minimal inflammation, while we used non-IC cystitis as the control. Thus up-regulation of the CXCR3 pathway might have reflected the common down-stream changes of chronic inflammation. On the other hand, increased expression of CXCR3 in infiltrating plasma cells is specific to HIC specimens.

CXCR3 receptor is expressed in several immune cells including mast cells, lymphocytes, plasma cells, and most preferentially activated T cells and T helper 1 cells^15^,^16^. The major physiological role of the CXCR3 pathway is chemotaxis of these immune cells into inflammatory sites^15^. Accumulated Th1 cells release IFN-ɣ, which stimulates production of CXCR3-binding chemokines (CXCL9, 10, and 11) from the resident cells at the inflammatory site^12^. Thus the CXCR3 pathway contributes to the development of chronic inflammatory reactions by creating local amplification loops.

In addition, chronic inflammation maintained by persistent, specific immune responses results in modulation of chemokine receptor expression on B cells and plasma cells^17^,^18^,^19^. It is well-recognized that CXCR3 expression of B cells and plasma cells is up-regulated in rheumatoid arthritis and systemic lupus erythematosus^20^. In this context, the observed increased CXCR3 expression of plasma cells in the HIC bladder may reflect the chronic activation and differentiation of B cells induced by particular antigens. This assumption is also supported by our previous report indicating frequent expansion of clonal B cells in HIC^9^, and by reports in the literature on the clinical association between IC and other autoimmune diseases, the presence of autoantibody in sera and urine of IC patients, and the experimental autoimmune cystitis model against bladder-specific uroplakin peptide^21^,^22^. Taken together with these reports, the current study results suggest expansion of a specific B cell population and its dominant role in the pathophysiology of HIC, as demonstrated in other immune response-related chronic inflammatory disorders^23^,^24^.

Due to the crucial role of the CXCR3 pathway, it has attracted attention as a therapeutic target with potential clinical application in various chronic inflammatory disorders^25^. In another autoimmune murine cystitis model using mice comparable to human IC, anti-CXCL10 antibody reduced the up-regulated level of CXCR3 and its ligands, and ameliorated the severity of cystitis^26^. Immunosuppressant therapies such as a steroidal, molecular-targeted therapy, or anti-chemokine therapy are potentially effective for HIC patients.

Limitations of the present study should be mentioned. The control samples, non-IC chronic cystitis, may not be optimal. Sampling bias cannot be excluded as a result of arbitrary selection. In addition, co-expression of CXCR3 and CD138 in lymphoplasmacytic cells was not confidently confirmed by flow cytometry analysis.

In conclusion, infiltration of CXCR3-positive plasma cells is a characteristic feature of HIC. The CXCR3 pathway and specific immune responses may be involved in accumulation/retention of plasma cells and pathophysiology of the HIC bladder.

## Materials and Methods

Ethical approval was obtained from the Institutional Review Board of The University of Tokyo (Reference No. 3124 and 2381). All the methods were carried out in accordance with the approved guidelines. Written informed consent was obtained from all patients.

### Tissue samples

A total of 54 bladder biopsy samples were taken from 27 patients with HIC during 2008 to 2011. Two samples were obtained from each of 27 HIC patients, one from the Hunner lesion and one from a non-lesion area, totalling 54. Samples diagnosed as chronic cystitis during 2009 to 2014 were retrieved from the archives of the Department of Pathology at the University of Tokyo Hospital, and histologically reviewed. Among them, we selected 23 samples from 15 non-IC patients, which showed, histologically, roughly the same degree of chronic inflammation as HIC specimens. We designated them the ‘non-IC cystitis’ group. Diagnosis of HIC was made according to the clinical guidelines for interstitial cystitis and hypersensitive bladder syndrome and European Society for the Study of IC/PBS criteria^1^,^6^. All the IC patients fulfilled the National Institute of Diabetes and Digestive and Kidney Diseases criteria^27^. Diagnosis of non-IC cystitis was made by histological evidence of chronic inflammation represented by predominant stromal infiltration of lymphoplasmacytic cells, edema, and fibrosis. Patients who had undergone intravesical administration of Bacillus Calmette-Guerin (BCG) or anti-cancer agents for bladder cancer were excluded.

### Immunohistochemistry

Serial 4-μm sections were used for immunohistochemistry (IHC) throughout. IHC staining was performed according to routine procedures on a Ventana Benchmark XT autostainer (Ventana Medical Systems, Tucson, AZ, USA).

We used the antibodies CD3 (1:50, Clone LN10; Novocastra, Newcastle upon Tyne, UK), CD20 (1:100, Clone L26, Dako, Glostrup, Denmark) and CD138 (prediluted, Clone B-A38, Nichirei Bioscience, Tokyo, Japan) to detect T-lymphocytes, B-lymphocytes, and plasma cells, respectively. Mouse monoclonal anti-CXCR3 antibody (1:100, Clone 1C6; BD Biosciences Pharmingen, Heidelberg, Germany) was used in this study. Appropriate control of each antibody was included.

### Quantitative analysis of the immunohistochemically stained cells

Images of stained slides were digitized by the NanoZoomer Digital Pathology system (Hamamatsu Photonics, Hamamatsu, Japan), followed by digital quantification using image analysis software (Tissue Studio^®^, version 3.5, Definiens AG, Munich, Germany)^8^. The number of CD3-positive T cells, CD20-positive B cells, CD138-positive plasma cells, lymphoplasmacytic cells (the sum of CD3-, CD20-, and CD138-positive cells), and CXCR3-positive cells were counted as described previously^9^. Furthermore, proportion of the number of CXCR3-and CD138-positive cells to the number of all mononuclear cells in randomly selected 3 “plasma cell-rich area”, defined as an area with more than a third of inflammatory cells as plasma cells in ×200 power fields^9^ were examined using adjacent sections in HIC and non-IC cystitis cases (if both specimens from the Hunner lesion and a non-lesion area were available from the same patient with HIC, one with more plasma cells was examined).

### Immunofluorescence

Double-immunofluorescence for CXCR3 and CD138 was used in selected specimens to further detect CXCR3 expression pattern in plasma cells. Mouse monoclonal anti-CXCR3 antibody described above was diluted at a concentration of 1:50, and rabbit monoclonal anti-human CD138 antibody (1:50, Clone SP152; LifeSpan BioSciences, Seattle, WA, USA) was used. Tissues were deparaffinized in xylene and graded ethanol. Antigen retrieval was performed in 10 mM EDTA buffer, pH 8.0 for 10 minutes in an autoclave oven. Incubation of the primary antibodies for 1 hour at room temperature was followed by incubation with Alexa Fluor 488-conjugated anti-mouse and Alexa Fluor 594-conjugated anti-rabbit secondary antibodies. Between incubation steps, the slides were rinsed with tris buffered saline. Isotype-matched immunoglobulins were used as negative controls.

### Correlation between cell numbers and clinical parameters in HIC cases

We explored the correlations between cell numbers and clinical parameters, including age, years from onset to biopsy, O’Leary and Sant’s symptom index and problem index (OSSI/OSPI), visual analogue scale for pain (VAS), urinary frequency, maximum voided volume, and the bladder capacity measured at biopsy.

### Statistical analysis

The Wilcoxon rank-sum test for two groups comparison and the Steel-Dwass test for multiple comparison were used for continuous variables, and Fisher’s exact test was used for categorical variables. The Spearman rank correlation coefficient test and logistic regression analysis were applied for the correlation between continuous variables and categorical variables, respectively. A *P-*value less than 0.05 was considered to be statistically significant. All statistical calculations were carried out with JMP^®^ Pro, version 11 (SAS institute, Cary, NC, USA).

## Additional Information

**How to cite this article**: Akiyama, Y. *et al*. Increased CXCR3 Expression of Infiltrating Plasma Cells in Hunner Type Interstitial Cystitis. *Sci. Rep.* **6**, 28652; doi: 10.1038/srep28652 (2016).

## Supplementary Material

Supplementary Information

## Figures and Tables

**Table 1:** Demographics of patients with Hunner type interstitial cystitis.

No. (male/female)	27 (3/24)
Mean age at the time of biopsy (years)	68.4 ± 11.4 [38–88]‡
Age at onset of IC (years)	65.1 ± 10.5 [38–80]
Years from symptom onset to biopsy (years)	3.3 ± 2.6 [0–8]
OSSI†	13.1 ± 4.1 [7–20]
OSPI†	11.4 ± 3.8 [3–16]
VAS†	6.4 ± 2.4 [1–10]
Urinary frequency (/day)	16.3 ± 5.7 [7–30]
Maximum voided volume (mL)	163.8 ± 59.6 [50–300]
Maximum bladder capacity at hydrodistension (mL)	521.2 ± 181.8 [200–900]

^†^OSSI/OSPI: O’Leary and Sant’s symptom index and problem index, VAS: visual analogue scale (for pain).

^‡^mean ± SD [range].

**Table 2:** Correlation among cell numbers in bladder biopsy samples.

(cells/mm^2^)	CXCR3-positive cells			CD3-positive T cells			CD20-positive B cells			CD138-positive plasma cells			Lymphoplasmacytic cells‡		
	HIC	HIC	Non-IC	HIC	HIC	Non-IC	HIC	HIC	Non-IC	HIC	HIC	Non-IC	HIC	HIC	Non-IC
	(L)†	(NL)	cystitis	(L)	(NL)	cystitis	(L)	(NL)	cystitis	(L)	(NL)	cystitis	(L)	(NL)	cystitis
CXCR3-positive cells				**0.34**	**0.40**	**0.51**	**0.58**	**0.48**	0.33	**0.48**	**0.47**	−0.11	**0.56**	**0.45**	**0.46**
				(0.04)	(0.04)	(<0.01)	(<0.01)	(0.02)	(0.07)	(0.01)	(0.01)	(0.62)	(0.01)	(0.01)	(0.02)
CD3-positive cells							**0.62**	**0.66**	**0.54**	**0.44**	**0.68**	0.22	**0.82**	**0.80**	**0.91**
							(<0.01)	(<0.01)	(0.01)	(0.02)	(<0.01)	(0.58)	(<0.01)	(<0.01)	(<0.01)
CD20-positive B cells										**0.43**	**0.41**	**0.40**	**0.91**	**0.82**	**0.76**
										(0.02)	(0.04)	(0.02)	(<0.01)	(<0.01)	(<0.01)
CD138-positive plasma cells													**0.68**	**0.72**	**0.34**
													(<0.01)	(<0.01)	(0.04)

^†^Number of samples: 27 for HIC (Hunner lesion: L), 27 for HIC (non-lesion area: NL) and 23 for Non-IC cystitis.

^‡^Lymphoplasmacytic cells: Sum of the CD3-positive cells, CD20-positive cells and CD138-positive cells.

^¶^Spearman’s correlation coefficient ρ and *P*-value (in parentheses), bold when *P* < 0.05.

**Table 3:** Proportion of the number of CD138-and CXCR3-positive cells to the number of all mononuclear cells in randomly selected 3 plasma cell-rich areas in HIC and non-IC cystitis cases^†^.

	CD138-positive cells/Mononuclear cells	CXCR3-positive cells/Mononuclear cells	(CD138-positive cells/Mononuclear cells) + CXCR3-positive cells/Mononuclear cells)
HIC (n = 22)	0.63 ± 0.15	0.44 ± 0.23	1.07 ± 0.24
Non-IC cystitis (n = 8)	0.51 ± 0.15	0.29 ± 0.14	0.80 ± 0.17
*P* value	0.17	0.10	0.04^*^

^†^HIC: Hunner type interstitial cystitis

^¶^mean ± SD

Significant difference: ^*^P < 0.01 by Wilcoxon rank-sum test.

**Table 4:** Correlation between cell numbers and clinical parameters in HIC cases^†^.

	CD3 (cells/mm^2^)		CD20 (cells/mm^2^)		CD138 (cells/mm^2^)		CXCR3 (cells/mm^2^)	
	HIC (L)	HIC (NL)	HIC (L)	HIC (NL)	HIC (L)	HIC (NL)	HIC (L)	HIC (NL)
	(n = 27)	(n = 27)	(n = 27)	(n = 27)	(n = 27)	(n = 27)	(n = 27)	(n = 27)
Age (years)	ρ = 0.02,	ρ = 0.04,	ρ = −0.04,	ρ = 0.04,	ρ = 0.35,	ρ = 0.19,	ρ = 0.18,	ρ = −0.20,
	*P* = 0.94	*P* = 0.84	*P* = 0.85	*P* = 0.84	*P* = 0.07	*P* = 0.35	*P* = 0.36	*P* = 0.31
Years from onset to biopsy (years)	ρ = 0.003,	ρ = −0.001,	ρ = 0.24,	ρ = −0.02,	ρ = 0.16,	ρ = 0.33,	ρ = 0.38,	ρ = −0.13,
	*P* = 0.99	*P* = 0.98	*P* = 0.23	*P* = 0.94	*P* = 0.42	*P* = 0.09	*P* = 0.05	*P* = 0.52
OSSI†	ρ = 0.05,	ρ = 0.31,	ρ = −0.11,	ρ = 0.37,	ρ = −0.19,	ρ = 0.34,	ρ = −0.30,	ρ = 0.25,
	*P* = 0.81	*P* = 0.13	*P* = 0.59	*P* = 0.06	*P* = 0.36	*P* = 0.09	*P* = 0.14	*P* = 0.22
OSPI†	ρ = 0.14,	ρ = 0.12,	ρ = 0.004,	ρ = −0.06,	ρ = −0.14,	ρ = 0.20,	ρ = −0.22,	ρ = 0.08,
	*P* = 0.50	*P* = 0.55	*P* = 0.98	*P* = 0.78	*P* = 0.51	*P* = 0.34	*P* = 0.28	*P* = 0.71
VAS†	ρ = 0.18 ,	ρ = 0.31,	ρ = 0.12,	ρ = 0.25,	ρ = −0.01,	ρ = 0.15,	ρ = 0.001,	ρ = −0.14,
	*P* = 0.37	*P* = 0.13	*P* = 0.54	*P* = 0.22	*P* = 0.94	*P* = 0.47	*P* = 0.99	*P* = 0.48
Urinary frequency	ρ = 0.22,	ρ = 0.37,	ρ = 0.04,	ρ = 0.30,	ρ = 0.03,	ρ = 0.37,	ρ = −0.22,	ρ = 0.37,
	*P* = 0.28	*P* = 0.06	*P* = 0.84	*P* = 0.14	*P* = 0.89	*P* = 0.06	*P* = 0.27	*P* = 0.06
Average voided volume (mL)	ρ = 0.10,	ρ = −0.16,	ρ = 0.21,	ρ = −0.22,	ρ = 0.12,	ρ = −0.40,	ρ = 0.16,	ρ = −0.43,
	*P* = 0.62	*P* = 0.43	*P* = 0.30	*P* = 0.29	*P* = 0.56	*P* = 0.07	*P* = 0.43	*P* = 0.06
Maximum voided volume (mL)	ρ = 0.07,	ρ = −0.05,	ρ = 0.25,	ρ = −0.11,	ρ = 0.01,	ρ = −0.19,	ρ = 0.06,	ρ = −0.18,
	*P* = 0.73	*P* = 0.81	*P* = 0.23	*P* = 0.59	*P* = 0.98	*P* = 0.35	*P* = 0.79	*P* = 0.37
Maximum bladder capacityat hydrodistension (mL)	ρ = −0.13,	ρ = 0.06,	ρ = −0.03,	ρ = 0.25,	ρ = −0.18,	ρ = −0.07,	ρ = 0.02,	ρ = −0.01,
	*P* = 0.53	*P* = 0.78	*P* = 0.89	*P* = 0.22	*P* = 0.38	*P* = 0.75	*P* = 0.91	*P* = 0.95

HIC (L): Hunner type interstitial cystitis-Hunner lesion, HIC (NL): Hunner type interstitial cystitis-non-lesion area, OSSI/OSPI = O’Leary and Sant’s symptom index and problem index, VAS = visual analogue scale (for pain).

Significant difference: ^*^P < 0.05 by Spearman rank coefficient correlation test.
