# Supplementary material for: Increased CXCR3 Expression of Infiltrating Plasma Cells in Hunner Type Interstitial Cystitis
Source: Sci Rep. 2016 Jun 24;6:28652. doi: 10.1038/srep28652 (PMC4919639; doi:10.1038/srep28652)
Supplement: Supplementary Information [file srep28652-s1.pdf]

Supplementary Information

Increased CXCR3 Expression of Infiltrating Plasma Cells in Hunner Type Interstitial Cystitis

Yoshiyuki Akiyama[1, 4], Teppei Morikawa[2], Daichi Maeda[3], Yukako Shintani[2], Aya Niimi[1], Akira Nomiya[5], Atsuhito Nakayama[2], Yasuhiko Igawa[4], Masashi Fukayama[2] and Yukio Homma[1]

[1] Department of Urology, Graduate School of Medicine, The University of Tokyo, Tokyo, Japan

[2] Department of Pathology, Graduate School of Medicine, The University of Tokyo, Tokyo, Japan

[3] Department of Cellular and Organ Pathology, Graduate School of Medicine, Akita University, Akita, Japan

[4] Department of Continence Medicine, Graduate School of Medicine, The University of Tokyo, Tokyo, Japan

[5] Department of Urology, Mitsui Memorial Hospital, Tokyo, Japan

Supplementary Table S1. Demographics and cell density in non-IC cystitis group associated with or without bladder cancer

	**Associated with bladder cancer** (N = 8, n = 12)	**Associated without bladder cancer** (N = 7, n = 11)	***P* value**
No. (male / female)	8 (5 / 3)	7 (3 / 4)	0.62
Mean age at the time of biopsy (years)	73.8 ± 10.8 [54 – 85]†	71.0 ± 9.5 [57 - 85]	0.56
CXCR3-positive cells (n/mm^2)	73.3 (40.7 – 152.6) ‡	146.1 (12.9 – 185.9)	0.78
CD3-postive cells (n/mm^2)	698.9 (354.0 – 1199.7)	353.0 (178.0 – 705.0)	0.07
CD20-positive cells (n/mm^2)	208.8 (112.0 – 366.1)	162.6 (23.3 – 250.0)	0.41
CD138-positive cells (n/mm^2)	56.8 (16.4 – 124.8)	49.0 (29.0 – 107.0)	1.00
Lymphoplasmacytic cells¶ (n/mm^2)	1066.1 (582.5 – 1627.1)	530.9 (348.4 – 1462.7)	0.09

† ‡ Values are expressed as mean ± SD [range] for age, or median (interquartile range) for cell numbers.

¶ Lymphoplasmacytic cells: Sum of the CD3-positive cells, CD20-positive cells and CD138-positive cells

Supplementary Table S2. Correlations among cell numbers in non-IC cystitis group associated with or without bladder cancer

(cells/mm^2)	CXCR3-positive cells		CD3-positive T cells		CD20-positive B cells		CD138-positive plasma cells		Lymphoplasmacytic cells‡	
	Associated with BC†	Associated without BC	Associated with BC	Associated without BC	Associated with BC	Associated without BC	Associated with BC	Associated without BC	Associated with BC	Associated without BC
CXCR3-positive cells			**0.56**¶ (0.05)	**0.55** (0.04)	0.32 (0.31)	0.35 (0.08)	0.07 (0.81)	-0.11 (0.75)	**0.60** (0.04)	0.58 (0.06)
CD3-positive cells					0.50 (0.10)	**0.61** (0.04)	0.34 (0.28)	-0.08 (0.81)	**0.97** (<0.01)	**0.93** (<0.01)
CD20-positive B cells							**0.63** (0.03)	-0.25 (0.45)	**0.64** (0.02)	**0.77** (<0.01)
Cd138-poshitivule plasma sells									0.39 (0.20)	-0.04 (0.92)

†Number of samples: 12 for patients with bladder cancer (BC), 11 for patients without BC

‡Lymphoplasmacytic cells: Sum of the CD3-positive cells, CD20-positive cells and CD138-positive cells

¶Spearman's correlation coefficient ρ and *P*-value (in parentheses), bold when *P* <0.05
